# Enhancement in the Physico-Mechanical Functions of Seaweed Biopolymer Film via Embedding Fillers for Plasticulture Application—A Comparison with Conventional Biodegradable Mulch Film

**DOI:** 10.3390/polym11020210

**Published:** 2019-01-26

**Authors:** Hasan M, E.W.N. Chong, Shima Jafarzadeh, M.T. Paridah, Deepu A. Gopakumar, H.A. Tajarudin, Sabu Thomas, H.P.S. Abdul Khalil

**Affiliations:** 1 Chemical Education Department, Universitas Syiah Kuala, Jln. Tgk. Daud Beureueh Darussalam Banda Aceh 23311, Indonesia; muhammadhasan.kimia@unsyiah.ac.id; 2 School of Industrial Technology, Universiti Sains Malaysia, 11800 Penang, Malaysia; ecarborea929@gmail.com (E.W.N.C.); deepu1789@gmail.com (D.A.G.); azan@usm.my (H.A.T.); 3 Food Biopolymer Research Group, Food Technology Division, School of Industrial Technology, University Sains Malaysia, 11800 Minden, Penang, Malaysia; shimajafar@yahoo.com; 4 Institute of Tropical Forestry and Forest Products (INTROP), Universiti Putra Malaysia, Serdang, Selangor, Malaysia; 5 International and InterUniversity Centre for Nanoscience and Nanotechnology, Mahatma Gandhi University, Kottayam-686560, Kerala, India; sabuthomas@mgu.ac.in

**Keywords:** bio-degradable polymer film, seaweed polymer, plasticulture application, conventional film, calcium carbonate

## Abstract

This study aimed to compare the performance of fabricated microbially induced precipitated calcium carbonate– (MB–CaCO_3_) based red seaweed (*Kappaphycus alvarezii*) bio-polymer film and commercial calcium carbonate– (C–CaCO_3_) based red seaweed bio-film with the conventional biodegradable mulch film. To the best of our knowledge, there has been limited research on the application of commercial CaCO_3_ (C–CaCO_3_) and microbially induced CaCO_3_ (MB–CaCO_3_) as fillers for the preparation of films from seaweed bio-polymer and comparison with biodegradable commercial plasticulture packaging. The results revealed that the mechanical, contact angle, and biodegradability properties of the polymer composite films incorporated with C–CaCO_3_ and MB–CaCO_3_ fillers were comparable or even superior than the conventional biodegradable mulch film. The seaweed polymer film incorporated with MB–CaCO_3_ showed the highest contact angle of 100.94°, whereas conventional biodegradable mulch film showed a contact angle of 90.25°. The enhanced contact angle of MB–CaCO_3_ resulted in high barrier properties, which is highly desired in the current scenario for plasticulture packaging application. The water vapor permeability of MB–CaCO_3_ based seaweed films was low (2.05 ± 1.06 g·m/m^2^·s·Pa) when compared to conventional mulch film (2.68 ± 0.35 g·m/m^2^·s·Pa), which makes the fabricated film an ideal candidate for plasticulture application. The highest tensile strength (TS) was achieved by seaweed-based film filled with commercial CaCO_3_ (84.92% higher than conventional mulch film). SEM images of the fractured surfaces of the fabricated films revealed the strong interaction between seaweed and fillers. Furthermore, composite films incorporated with MB–CaCO_3_ promote brighter film, better water barrier, hydrophobicity, and biodegradability compared to C–CaCO_3_ based seaweed polymer film and conventional mulch film. From this demonstrated work, it can be concluded that the fabricated MB–CaCO_3_ based seaweed biopolymer film will be a promising candidate for plasticulture and agricultural application.

## 1. Introduction

Due to the extremely harmful ecological effects—especially environmental pollution—modern, biodegradable, effective, and environmentally-friendly materials have been offered to strengthen the quality and efficiency of the production of crops in the agricultural sector, and to replace traditional petrochemical plastics, decrease pesticide use, and ease the harvesting process [1,2]. In the past years, the majority of the conventional plasticultures were made from synthetic materials, but the use of synthetic plastic takes a long time to decompose in nature. If synthetic plastics are burnt, it releases harmful gases into the atmosphere and can result in environmental pollution, and when buried, plastic in soil is difficult to decompose. Reference [3] believes that the global demand for plastic film, particularly in the agricultural sector, is going to rise by 69%, from 4.4 million tons in 2012 to 7.4 million tons by the year 2019. This concern has caused a shift in using biodegradable polymers rather than synthetic materials.

Biopolymers that are taken from natural resources, such as starch, seaweed, lignin, cellulose, protein, and chitin, could easily degrade as they are exposed to bioactive environments, such as compost and soil [4,5,6]. Biopolymers are vastly utilized for different purposes, such as fertilizer, food, biomedical, fodder, drug, delivery, packaging, regenerative medicine, heavy metal sorption, biodegradability, and biocompatibility [7,8,9,10].

Seaweed is a vital resource for natural biopolymers, which has been widely employed around world. The main seaweed derivatives, such as carrageenan and alginate, have been broadly used in the agricultural industry, packaging, cosmetics, food, and pharmaceuticals, since they are easily accessible with high organic content and impressive phycocolloids and biodegradability; they are also known as an effective gas barrier with mechanical properties [11]. Seaweed phycocolloids are reported to have a colloid system as they are exposed to water, either in solubilized particles or a gel form. Nevertheless, they are known to be hydrophilic in nature. Therefore, they show poor thermal stability, water barrier properties, and mechanical properties. As a result, experts made a great effort to modify and promote biopolymer properties via blending or grafting with other types of polymers or integrating fillers to reinforce their competitiveness by means of the commodity polymers [10,12]. Literature abounds with evidence reflecting the significance of using organic fillers to increase the functional properties of raw seaweed film [2,13]. Further, it is also possible to use inorganic fillers to promote the properties of the raw seaweed film, as past research has successfully used inorganic fillers, such as nano-clay and montmorillonite clay (MMT), calcium carbonate (CaCO_3_), and silver nanoparticles into carrageenan- and alginate-based films [14,15].

Calcium carbonate (CaCO_3_) is one of the cheapest and most abundant minerals on earth, which in agriculture is used as fertilizer to supply calcium to plants, stabilize pH values, and decrease acidic conditions in the soil. The previous studies have stressed the importance of incorporating CaCO_3_ in polymer and biopolymer materials in order to improve the thermal, barrier, and mechanical properties of synthetic polymer matrices [16,17]. This natural mineral material is usually derived from excavating carbonate-containing rock. However, using such a method to obtain CaCO_3_ could contribute to water, air, and sound pollution, which are unsustainable for long periods of exposure. Therefore, in order to decrease the environmentally adverse effects, a green and sustainable method of microbial induced calcite precipitation (MICP) could be a great substitution to the conventional method to gain highly purified CaCO_3_ in a short time. This approach is helped by ureolytic bacteria, such as *Bacillus cereus, Bacillus pasteurii*, and *Bacillus sphaericus*, which increase the precipitation of CaCO_3_ under intense calcium environments. It is easy to control the obtained CaCO_3_ by considering several environmental parameters, such as bacteria cell concentration, bacteria types, pH, temperature, calcium, and urea concentrations [18]. Previous research has shown CaCO_3_ use by MICP method in numerous applications, such as plastic, cement, rubber fluorescent particles in fluorescent markers, and stationery ink [19]. 

Until now, there has been limited research on the application of commercial CaCO_3_ and microbially induced CaCO_3_ as fillers for the preparation of films from seaweed, and limited comparison with commercial biodegradable packaging. Hence, the current study intended to fabricate high-performance seaweed-based films incorporated with commercial CaCO_3_ and microbially induced CaCO_3_ to enhance their functional properties, and then compare these with commercial biodegradable packaging. In addition, a characterization study of these CaCO_3_ fillers was also determined and compared with commercial biodegradable packaging.

## 2. Materials and Methods

### 2.1. Materials

The films were fabricated using Analytical grade glycerol (Univar Inc., Redmond, WA, USA) as a plasticizer, CaCO_3_ as an inorganic filler, and raw red seaweed (Kappaphycus alvarezii), which was purchased from Green leaf Synergy Sdn. Bhd. (Tawau, Sabah, Malaysia). The chemical composition of Kappaphycus alvarezii were 1.10% lipid, 11.57% ash, 65.20% carbohydrate, and 3.40% of protein content. The growth media, such as synthetic urea (Bendosen Laboratory Chemicals, Bendosen, Norway) and yeast extract (HiMEDIA, Mumbai, India), were used in order to culture *Bacillus sphaericus* (LMG 22,557, city, Brazil). The precipitation of CaCO_3_ was carried out using nitrate Ca(NO_3_)_2_.4H_2_O (Bendosen Laboratory Chemicals, Bendosen Norway).The reference material was the commercial CaCO_3_ (C–CaCO_3_), which was supplied by ChemPur (Selangor, Malaysia). Commercial biodegradable film, known as PLA (Polylactic Acid) mulch, was purchased directly from a manufacturing company in China (Changzhou Greencradleland Macromolecule Materials CO., LTD), which was processed by blow molding method.

### 2.2. Microbially Induced Calcium Carbonate Precipitation (MICP)

Based on the method proposed by [20], MICP was performed with a modest modification. The present study used the bacterium strain *Bacillus sphaericus* LMG 22,557 to create microbially induced CaCO_3_ (MB–CaCO_3_). The bacteria were stored as a stock culture at 80 °C. First, the stock culture of *Bacillus sphaericus* LMG 22,557 was thawed and recovered. In the activation stage, the bacteria were subcultured into a new growth medium of 10% inoculum and incubated for 24 h at 30 °C and 100 rpm. The combination of yeast extract and synthetic urea was considered as the growth media in order to culture *Bacillus sphaericus* with a composition of 20 g/L yeast extract and 20 g/L urea solution. A 0.45 mm filter was used to filter the urea solution, which was then sterilized. Both yeast and urea extract solutions were separately sterilized using an autoclave for 15 min at 120 °C prior to mixing them together. Before fermentation, subculture was carried out at 30 °C and 100 rpm in the shaker flask, which contained a urea growth medium. It was incubated until the bacteria achieved exponential growth phase. Fermentation began as 720 mL of urea growth medium was inoculated with 80 mL of *Bacillus sphaericus* inoculums in a 2 L shaker flask. It was then incubated at 30 °C and 100 rpm. As soon as the stationary growth phase was achieved, the fermentation was stopped. At the final stage, the cells were harvested and taken away by centrifugation (Kubota, Tokyo, Japan) at 6000 rpm for 10 min. Then, calcium nitrate Ca(NO_3_)_2_·4H_2_O was incorporated into the sample equivalent to the bicarbonate ion concentration, which was measured by alkalinity test. As the mixing was settled, the CaCO_3_ precipitation was formed and oven-dried at 70 °C overnight.

### 2.3. Preparation of MB–CaCO_3_/Seaweed and C–CaCO_3_/Seaweed Composite Films

This study employed a simple solution casting method to prepare the composite films. The residues and excessive dirt were removed from the red seaweed (Kappaphycus alvarezii). Afterwards, the clean seaweed was chopped and dried in an oven at 40 °C for 3 days [2]. This study used raw seaweed as a base matrix without an additional extraction step. Next, 4.0 g (oven-dried weight) of clean raw seaweed in 200 mL of distilled water with 2.0 g of glycerol as plasticizer was dissolved on a heating plate, in order to prepare a composite solution. According to the dry weight of the seaweed (wt %), MB–CaCO_3_ and C–CaCO_3_ were added into the solution with different loadings (0%, 0.08%, 0.1%, 0.15%, 0.2%). Heating the solution at 90 °C for 1 h on a hot plate magnetic stirrer at 150 rpm made it completely solubilized (IKA® 11 C-MAG HS-7, Selangor, Malaysia).The solution was then placed on a casting tray (20 × 20 cm^2^) and put in a ventilated oven at 40 °C for 24 h. Prior to testing, all peeled-off films were further conditioned in a chamber at 25 ± 0.5 °C and 50% relative humidity (RH). At least three replicates were developed for each single experiment. 

### 2.4. Characterization Studies

#### 2.4.1. Thickness

A precision digital micrometer was employed (Mitutoyo, Kanagawa, Japan) to the closest 0.001 mm at 5 random spots on each of the films in order to determine the film thickness, and the mean value was measured for each film. The measurements of barrier and mechanical properties of each sample were shown based on the mean thickness values. 

#### 2.4.2. Contact Angles

The sessile drop method via Contact Angle Analyzer (KSV CAM 101; KSV Instruments Ltd., Helsinki, Finland) under ambient temperature was used to measure the interfacial tensions of solid-liquid (wetting film properties). A syringe with 5 mL of water was released to the film surface. Images were immediately shot and documented after the water was released onto the film surface. The angle between the tangent and the baseline at the drop boundary was measured. Three measurements were recorded at different positions on the films in order to calculate the mean value. 

#### 2.4.3. Water Vapor Permeability

The modified ASTM E-96 1996 method was used to measure water vapor permeability. Based on the cup area (43 cm^2^), composite films were cut into a circle. The permeability cups with 30 mL of distilled water were placed over the cups and capped with the chosen films, performed in a controlled humidity chamber exposed to a temperature of 25 °C and 50% RH for six hours. Every hour the changes in water weight were documented. Testing was carried out three times for each single film formulation. The changes in cup weight versus time was graphically plotted and measured in linear regression (R2 > 0.95). The following equation was used to determine water vapor transmission rate (WVP):

(1) $$\mathrm{WVP}=\frac{\mathrm{WVTP}\times D}{\mathrm{Ps}\times \left(\frac{\Delta \mathrm{RH}}{100}\right)}\;\mathrm{WVTR}:\;\mathrm{Slope}/\mathrm{area}\;D:\;\mathrm{Film}\;\mathrm{thickness}\;\mathrm{Ps}:\;\mathrm{The}\;\mathrm{saturation}\;\mathrm{vapor}\;\mathrm{of}\;\mathrm{water}\;\mathrm{at}\;\mathrm{room}\;\mathrm{temperature}\;\mathrm{RH}:\;\mathrm{Relative}\;\mathrm{humidity}$$

#### 2.4.4. Optical Properties

According to [2], the opacity and color of the composite films were measured, employing a Data Color 400 Bench-Top Spectrophotometer programmed with Data color Match software (Data Color International, Lawrenceville, GA, USA) based on the CIE L*a*b* color scale. The film was put on a standard white surface plate to achieve the color coordinates of yellowness/blueness (*b*_standard_*) = 5.38, redness/greenness (*a*_standard_*) = 0.88, and the degree of lightness/brightness (*L*_standard_*) = 93.38. The measurements were carried out 3 times to gain a mean result per sample. The total difference in color (DE) and the chroma (C*) were calculated by Data Color Tools. Opacity was measured as the contrast ratio between the opacity of each film, both on the white standard and on the black standard. The results were reported in percentage (%). Three replicates were produced for each single film formulation.

#### 2.4.5. Mechanical Properties

Percentage of elongation (% E), modulus of elasticity (YM), and Tensile strength (TS) were measured based on the ASTM D-882-02, 2002 standard with slight modification. Films were chopped into strips of 10 cm length × 1 cm width; they had 5 replicates for each single film. The tensile tester MT1175 (Dia-Stron Instruments, UK) was equipped with 5 kg load cell in a controlled room at 25–28 °C, and 50% ± 5% RH was used to test the films. The first gauge length was specified at 100 mm and the crosshead speed was set at 50 mm/min.

#### 2.4.6. Thermal Properties

The residual (%), the maximum temperature (*T*_max_) of decomposition, and the onset temperature (*T*_on_) were measured via Thermogravimetric Analysis (TGA), relying on a Mettler-Toledo thermogravimetric analyzer model TGA/DSC 1, Switzerland. Roughly 10 mg of films received heat from 30 to 800 °C under nitrogen atmosphere at a heating pace of 10 °C/min. The residue was shown as the residual in percentage (%) after losing weight at 800 °C. The melting enthalpy, melting temperature (*T*_m_), and the onset of melting temperature (*T*_onset_) were determined by Perkin-Elmer differential Scanning Calorimetric (DSC) model 6, Switzerland. Nearly 10 mg of films were chopped into small pieces and packed in aluminum pans. The empty pan was considered as reference. The heating process of the films ranged from 30 to 350 °C under nitrogen atmosphere at a scanning rate of 10 °C/min.

#### 2.4.7. Structural Analysis-Fourier Transform Infrared (FTIR)

Concerning the chemical analysis, FTIR spectrophotometer (Perkin-Elmer, PC1600, Winter Street Waltham, MA, USA) was used to determine the chemical functional groups in attenuated total reflectance (ATR) mode. The absorbance spectrum produced in the range between 400 and 4000 cm^−1^ for each film was documented. Prior to FTIR analysis, the prepared films were cut in the size of 2 cm × 2 cm; they were then oven-dried at 60 °C for 24 h. 

#### 2.4.8. Morphological Property-Scanning Electron Microscope (SEM)

Before examining the surface morphology, the oven-dried films with a size of 1 cm × 1 cm were put-on double-sided Scotch tape; they were then coated with a thin gold (Au) layer by means of sputter coater Polaron SC515. The fractured morphology and surface of the films were recorded under SEM (EVO MA 10, Carl-ZEISS SMT, Oberkochen, Germany). To examine the films’ surface, 500× magnifications were used, while 2000× magnifications with 20 kV accelerated voltage were used to determine the films’ fracture (cross-section).

#### 2.4.9. Soil Burial Test

As suggested by [21], the composite film degradation was measured by weight loss during soil burial with slight modifications. Samples with 3 cm × 3 cm dimensions were placed under 4 cm of soil at ambient temperature; they were maintained at nearly 36% (*w*/*w*) moisture content. The composite films were dug out from the soil after 0, 10, 20, and 30 days of degradation. They were deliberately cleaned, and the weight loss differences were measured using the following equation:

(2) $$\mathrm{Weight}\;\mathrm{loss}\;(\%)=[\frac{\left(W1-W2\right)}{W1}]\;\times \;100\;W1=\mathrm{Initial}\;\mathrm{dry}\;\mathrm{weight}\;\mathrm{of}\;\mathrm{composite}\;\mathrm{film}\;W2=\mathrm{dry}\;\mathrm{weight}\;\mathrm{of}\;\mathrm{composite}\;\mathrm{film}\;\mathrm{after}\;\mathrm{cleaning}$$

#### 2.4.10. Statistical Analysis

One-way analysis of variance (ANOVA) using DSAASTAT ver.1.101 by Andrea Onofri was used to analyze the data of mechanical and physical properties. The significant differences (*p* < 0.05) among mechanical and physical properties of composite films and the control film were measured using a Post Hoc multiple comparisons test, which was performed by Tukey′s HSD.

## 3. Results and Discussion

### 3.1. Characterization of Composite Films

The physical and mechanical characteristics of selected seaweed-based films were compared to the conventional mulch film to evaluate their potential and competitiveness in application. The selected seaweed-based films included the control (seaweed-based film without filler), the best physical and mechanical properties of seaweed-based film filled with 0.1% commercial CaCO_3_, and the best physical and mechanical properties of seaweed-based film filled with 0.15% microbial-induced CaCO_3_ from our previously published work [2]. 

#### 3.1.1. Thickness

Film thickness is the basic characteristic necessary to be determined before proceeding to other testing, as the value is considered in determining mechanical strength and water barrier properties. As for agricultural purposes, film thickness as low as 5 µm is adequate to control weed growth in the field [22]. Thickness of seaweed-based composite films and conventional mulch films was measured using a precision digital micrometre (Mitutoyo, Kanagawa, Japan) with accuracy closest to 0.001 mm. Five measurements were made at random spots for each sample and the mean values were determined. The measurements of barrier and mechanical properties of each sample were shown based on the mean thickness values. As presented in Table 1, all the films were above 5 µm, with the highest film thickness being 61.2 µm, achieved by film filled with commercial CaCO_3_, while the lowest was 13.4 µm, achieved by conventional biodegradable mulch film. Based on the finding of Liu et al. (2013), there were no significant differences between the different thicknesses of high-density polyethylene (HDPE) films on weed growth and yield of paddy rice. Similarly, there were no significant differences (*p* < 0.05) found in the composite films by [2]. However, in the current study, results showed that there were significant differences between the four types of films from control to conventional biodegradable mulch film. This could be due to different materials and contents made up in the films, and film thickness is dependent on film composition and processing parameters [23]. 

#### 3.1.2. Contact Angles (CA)

**Figure 1:**
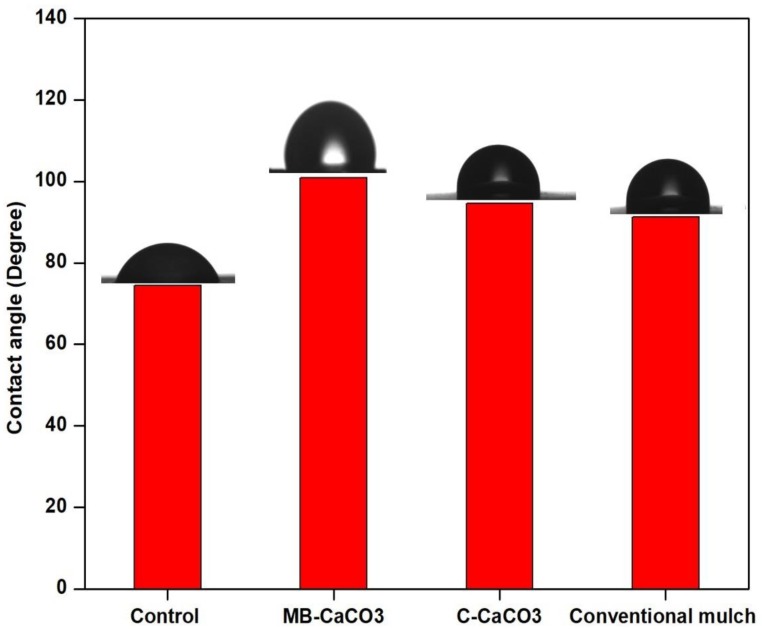
Contact angle of control, MB–CaCO_3_, C–CaCO_3_, and conventional mulch films.

Wettability is reported as high, as the contact angle is found to be lower than 90°, and vice versa [23]. Figure 1 shows that the contact angles (CA) of MB–CaCO_3_ based seaweed film, C–CaCO_3_ based seaweed film, and conventional mulch films were above 90°, except for control film. This interesting result indicated that seaweed-based biodegradable film incorporated with MB–CaCO_3_ and C–CaCO_3_ exhibited superior wetting resistance compared to the conventional biodegradable mulch film. The film filled with microbial induced CaCO_3_ denoted the highest CA (100.94°), while conventional biodegradable mulch film denoted the lowest CA (90.25°). Results verified that the fillers were able to improve film CA. Similar behavior was also found in carrageenan film with incorporation of other inorganic fillers, such as clay and silver nanoparticles (AgNPs), where the incorporation of inorganic fillers had proven to enhance CA of the neat films [24,25]. Furthermore, higher CA value also means better surface solid hydrophobicity. In this case, seaweed-based film filled with fillers showed better surface hydrophobicity compared to the control and the conventional biodegradable mulch film. This could be attributed to the higher hydrophobicity of fillers in the films, which causes the improvement of hydrophobicity of the seaweed-based films filled with fillers, as verified in [2]. 

#### 3.1.3. Water Vapor Permeability

Water vapor permeability (WVP) is determined to evaluate the water barrier properties of films. As shown in Table 1, the WVP of neat seaweed film decreased more by the incorporation of MB–CaCO_3_ fillers compared to C–CaCO_3_ fillers, as it is believed that the spherical shape and nano-size of MB–CaCO_3_ fillers are able to fill up the voids, hence creating a smoother surface and more structural layers in the morphology than the irregular shape and larger size of C–CaCO_3_. This phenomenon was validated by SEM observation in our previous publication [2]. According to [26], this behavior was due to the formation of torturous pathway against the migration of water molecules via the film, as filler was incorporated. Similar results have been reported for nanocomposite protein films [27,28]. As shown in the Table 1, the WVP of control, MB–CaCO_3_, C–CaCO_3_, and conventional mulch films were 3.87 ± 0.05^c^, 2.05 ± 1.06^a^, 2.45 ± 0.05^b^, and 2.68 ± 0.35^c^, respectively. The WVP is high for control seaweed film compared to other films, which was due to the hydrophilic nature of seaweed bio-polymer film, as shown in the Figure 1. In this case, it can be observed that the MB–CaCO_3_ based seaweed polymer film promoted better water barrier properties compared to all other films. It can be concluded that the MB–CaCO_3_ could be a promising candidate for plasticulture applications.

#### 3.1.4. Mechanical Properties

Mechanical properties are equally important as water barrier properties to determine film performance, especially in packaging and plasticulture. Mulch film should have enough strength, as it is usually installed on the soil by machines. Hence, weak mechanical properties are not desired to cater for certain stress and deformation during handling and fixing of the films on the soil. The mechanical characteristics, such as tensile strength (TS), elasticity modulus (E), and elongation at break points (EAB) of composite films based on seaweed and mulch films are summarized in Table 1. The results show that there are significant differences between the seaweed based-films and the conventional mulch film. Interestingly, seaweed-based films showed higher tensile strength (TS), Young’s modulus (YM), and elongation at break (EAB) compared to the conventional mulch film; furthermore, the addition of MB–CaCO_3_ and C–CaCO_3_ enhanced the mechanical properties of seaweed-based films. The highest TS was achieved by seaweed-based film filled with commercial CaCO_3_ (84.92% higher than conventional mulch film), followed by seaweed-based film filled with microbial-induced CaCO_3_ (82.14% higher than the conventional mulch film), control (72.73% higher than the conventional mulch film), and the conventional mulch film. Similarly, the highest YM was achieved by seaweed-based film filled with commercial CaCO_3_ (54.73% higher than conventional mulch film), followed by seaweed-based film filled with microbial-induced CaCO_3_ (44.45% higher than the conventional mulch film), control (12.89% higher than the conventional mulch film), and the conventional mulch film. 

As for EAB, the highest EAB was achieved by seaweed-based film filled with microbial-induced CaCO_3_ (46.19% higher than the conventional mulch film), followed by seaweed-based film filled with commercial CaCO_3_ (37.90% higher than conventional mulch film), control (32.67% higher than the conventional mulch film), and the conventional mulch film. This indicated that seaweed-based films possessed higher mechanical strength, rigidity, and flexibility, as well as were able to sustain higher load or stress compared to the conventional mulch film. The higher strength of seaweed-based films than the conventional mulch film can be attributed to the composition of seaweed-based film, where carrageenan found in red seaweed is able to contribute in forming strong film with better gelation properties [2]; moreover, an increase in mechanical properties with enhanced filler was reported to gain a desirable interfacial adhesion between matrix and fillers because of strong intermolecular interaction, which was facilitated by hydroxyl groups between red seaweed and CaCO_3_ [29]. The improved mechanical properties also suggested that CaCO_3_ and red seaweed had gained a desirable compatibility between one another because of fillers’ good dispersion in the matrix. TS and EAB for seaweed based-films were comparable to starch-based film, polyacrylate film, and poly (vinyl alcohol) film incorporated with natural fibers prepared by [30]. In fact, the TS and EAB in this study were higher than the biodegradable mulch films prepared by [30], with the TS ranging from 2.92 to 3.69 MPa and EAB ranging from 10.92% to 24.77%. In addition, the TS, E, and YM of these seaweed-based composite films were higher than the biodegradable mulch films fabricated using cassava starch [31].

#### 3.1.5. Color and Opacity Properties

Optical properties are important particularly for agricultural mulch film. The possible reason is that the various colors of mulch films indicate various radiation patterns in the canopies of crops, where the light reflectivity subsequently impacts the growth of the plant, the response, and the development of insects towards the plant [32]. Table 2 shows a comparison of physical and mechanical properties of some reported seaweed-based films for packaging applications, and it was clearly evident that the physical and mechanical properties of the fabricated seaweed-based films incorporated with C–CaCO_3_ and MB–CaCO_3_ were comparable to those of the previous results. 

Color and opacity tests are common to assess the color tone and opacity of a film by light-transmittance, where (L*) represents the lightness, (a*) represents the redness, (b*) represents the yellowness, and (C*) represents the chrome of the films. From Table 3, it can be observed that the conventional mulch film gained the highest lightness (L*). In other words, the conventional mulch film was brighter compared to all other seaweed-based films. Seaweed-based films showed higher values in redness, yellowness, and chrome. Seaweed-based films showed darker color because of the carotenoid pigment in the seaweed, which gives a red color to the seaweed. Furthermore, it can absorb light wavelength, thus, reducing the light rays from penetrating across the films [2]. On the other hand, the highest opacity of the films was achieved by seaweed-based film filled with commercial CaCO_3_. This means seaweed-based films appeared to be opaquer compared to the conventional mulch film. According to [37], clearer mulch film was not ideal for controlling weeds. However, it is preferred in the early stage to warm the soil, while brown plastic mulch provided the best combination of soil warming and weed control. Nevertheless, the color of the films can be custom-made to suit different crop production and purposes. In this study, seaweed-based films are close to brown color. This shows that there is potential for seaweed-based films to control weeds and warm the soil the same time.

#### 3.1.6. Thermal Properties

**Figure 2:**
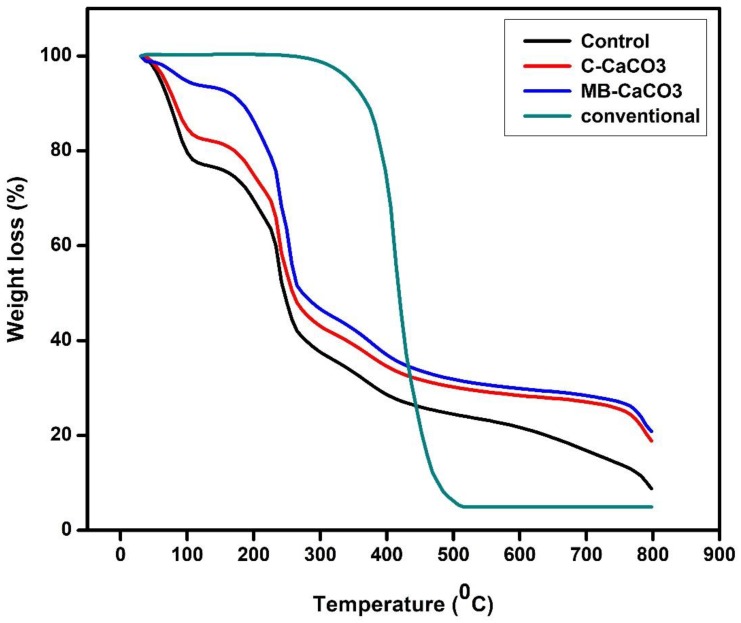
Thermogravimetric analysis (TGA) curves of control, C–CaCO_3_ based seaweed, MB–CaCO_3_ based seaweed, and conventional films.

From Figure 2 and Table 4, it is shown that the highest *T*_on_ was achieved by the conventional mulch film made of PLA, which was 260 °C. This result falls in the range of previous reports, from 335 to 339 °C [38], and from 270 to 373 °C [39]. *T*_on_ and *T*_max_ of the conventional mulch film were higher compared to the seaweed-based films, exhibiting better thermal stability than the seaweed-based films. However, with incorporation of MB–CaCO_3_ and C–CaCO_3_, the initial temperature of degradation (*T*_on_) and the maximum temperature of degradation (*T*_max_) were shifted to higher temperatures. This indicated that fillers improved the thermal stability of the red seaweed film. This could be attributed to their strong intermolecular interactions between seaweed matrix and fillers, which required more energy to break the intermolecular bonding, and thereby led to higher *T*_on_ and *T*_max_. The weight loss of residues (%) of the conventional mulch film was lower than those of seaweed-based films. This could be due to other residuals, such as mineral and CaCO_3_ content in seaweed-based films. Besides, the results were also in agreement with the results of the soil burial test, where the conventional mulch film did not show much degradation and weight loss after soil burial compared to seaweed-based films (Figures 5 and 6). 

#### 3.1.7. FTIR-ATR Analysis

**Figure 3:**
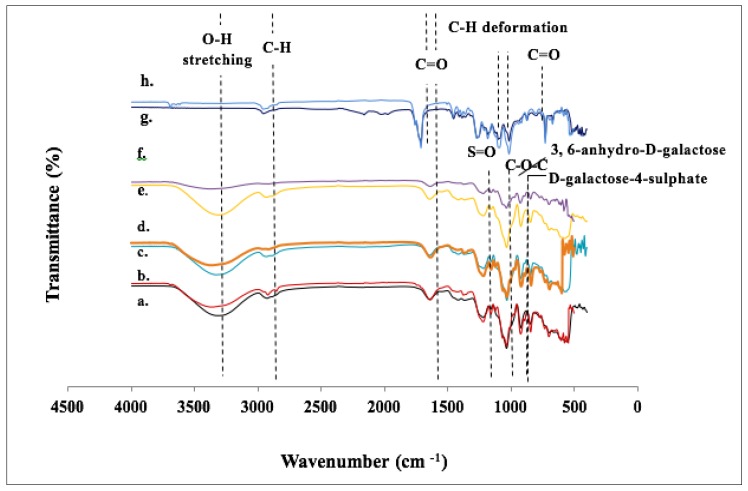
Fourier transform infrared (FT-IR) spectra of seaweed-based films filled with fillers and conventional mulch film before and after soil burial.

Figure 3 depicts the IR spectra of seaweed-based films and the conventional mulch film before and after soil burial of 6 months in between wavenumbers of 500 to 4000 cm^−1^. As reported by [2], seaweed-based films consist of broad and wide characteristic peaks in the range of 3320 to 3329 cm^−1^; these peaks corresponded to the presence of hydroxyl groups caused by O–H stretching vibration, which is due to the hydrophilicity nature of seaweed. Nevertheless, the incorporation of commercial and microbial-induced CaCO_3_ revealed that there was a reduction in peak intensity when compared to the control. The authors explained that this behavior occurred due to the strong interaction between seaweed and CaCO_3_, which was further supported by the enhancement of the physical and mechanical properties of the film when CaCO_3_ was incorporated. When comparing seaweed-based films with the conventional mulch film, it was obvious that there was no functional group found in the region of the O–H stretching vibration for the conventional mulch film. Besides the O–H stretching, the symmetric and asymmetric stretching of aliphatic C–H from CH_2_ group can be found in seaweed-based films ranging 2924 to 2889 cm^−1^, as well as conventional mulch film at 2959 cm^−1^, where a peak can also be found in PLA [40,41]. Absorption peaks ranging from 1643 to 1647 cm^−1^ were depicted in seaweed-based films, while the main peak found in conventional mulch at 1712 cm^−1^ was assigned to carbonyl group, as shown in the Table 5. This was caused by the stretching vibration of (C=O). Peaks at 1454 and 1411 cm^−1^ are corresponded to bending vibration of C–H group in seaweed-based the films [33], while absorption bands appearing at 1361 and 1386 cm^−1^ in conventional mulch film are attributed to CH deformation and asymmetry [41]. Absorption bands distinguished at 1210 and 1270 cm^−1^ correspond to the asymmetric stretching vibration of the S=O, which can be found in carrageenan (sulphated polysaccharide in red seaweed), while C-O stretching modes of the ester group appear at 1269 cm^−1^ in the PLA found in conventional mulch film. The peak at 1034 cm^−1^ represents the stretching vibration of C–O–C glycosidic bonds in the polysaccharides of seaweed-based films, while the C–O–C asymmetric mode appearing at 1016 cm^−1^ can be identified in the PLA found in conventional mulch film. Distinct peaks at 921.97 cm^−1^ and 844.82 cm^−1^ found in seaweed-based films indicate the presence of 3,6-anhydro-d-galactose and d-galactose-4-sulphate, respectively. According to [41], PLA is rich in carbon chains. Hence, another stretching vibration of C=O can be observed at 669 to 729 cm^−1^.

Seaweed-based films and conventional mulch film were compared after burial in soil for 6 months. It was noticed that all films showed reduction in the hydroxyl band (3500–3100 cm^−1^) due to degradation of organic compounds—mainly the seaweed. Apparently, the control film showed greater changes in the hydroxyl group compared to the other seaweed-based films filled with fillers. This occurrence could be due to the filler content in seaweed film, which caused them to compromise the rate of biodegradation. It is further clarified and supported by the soil burial test, based on percentage of weight loss (Figure 6). Reference [42] also reported the same phenomenon seen in CaCO_3_ was incorporated into starch-based film. It was obvious that seaweed-based films tend to show more changes, particularly in peak height reduction in hydroxyl group as compared to the conventional mulch film, as seaweed-based films are hydrophilic. 

#### 3.1.8. Morphological Test-Scanning Electron Microscope (SEM)

**Figure 4:**
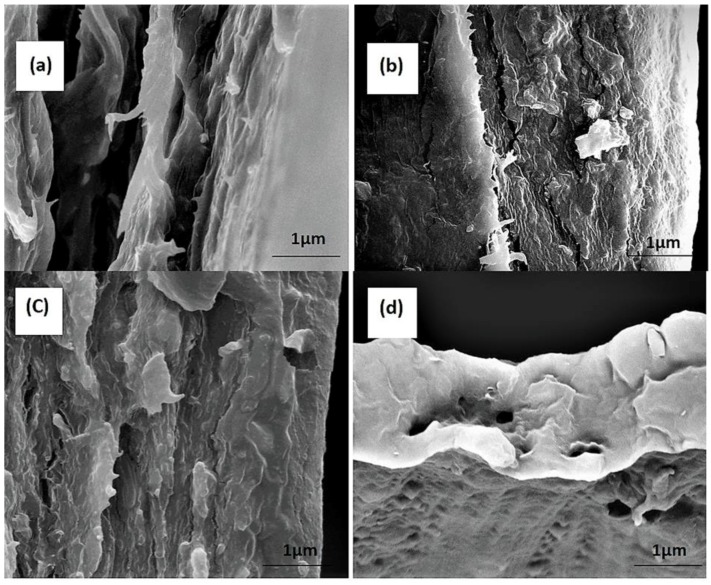
Fractured morphologies of (**a**) the control film and composite film with (**b**) 0.1% C–CaCO_3_; (**c**) 0.15% MB–CaCO_3_; and (**d**) conventional mulch film.

SEM images displayed in Figure 4 present the fractured surfaces of seaweed-based films and conventional mulch film obtained after a mechanical test. The fractured surfaces of seaweed-based films incorporated with 0.1% of commercial CaCO_3_ and 0.15% of microbial-induced CaCO_3_ showed a more compact morphology with lesser cavities compared to the fractured surface of control film. This suggests a strong interaction occurred between the seaweed and fillers, which could be due to the formation of strong intermolecular interaction, resulting in good interfacial stress transfer, which is also supported by the mechanical and physical tests, whereby the incorporation of CaCO_3_ led to enhancement in physical and mechanical properties of the neat seaweed film, indicating that the fillers were well-dispersed in the matrix. Meanwhile, composite films filled with CaCO_3_ were coarser and rougher, indicating strong interaction between C–CaCO_3_ and the matrix. Besides, C–CaCO_3_ itself contained a higher crystallinity percentage than MB–CaCO_3_, which increased the strength of the composite film; in addition, composite films filled MB–CaCO_3_ showed more organized layers as compared to composite films filled C–CaCO_3_. This could be attributed to the uniform size and shape of MB–CaCO_3_. A similar case can be observed in the starch-based film filled with CaCO_3_ [43]. Comparing seaweed-based films with the conventional mulch film, some holes can be observed from the morphology of conventional mulch film, suggesting weaker mechanical properties than that of seaweed-based films. 

#### 3.1.9. Biodegradability: Soil Compost Test

**Figure 5:**
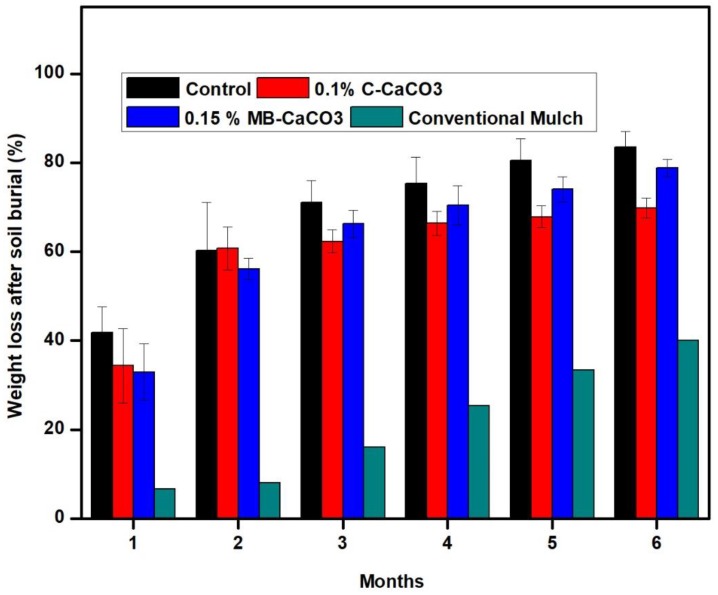
Weight loss changes (%) for 6-month soil burial test.

The soil compost test was extended to 6 months from our previously published work [2]. The changes of weight before and after exposure in soil were determined in percentage and plotted in graphs, as shown in Figure 5. 

Based on our previous work, biodegradation occurred even after 10 days of exposure in soil. The control (without CaCO_3_ fillers) was easily degraded since organic polymer is usually prone to microorganism attack, which was expected in this case [2]. This result was in agreement with the study done by [42] on starch/nano CaCO_3_ bio-nanocomposites with increasing filler loading. In general, the neat seaweed-based film showed the highest weight loss compared to the seaweed-based films filled with 0.1% of commercial CaCO_3_, 0.15% microbial-induced of CaCO_3_, and conventional mulch film. When compared between the composite films comprised of C–CaCO_3_ and MB–CaCO_3_, it was noticed that the weight loss for composite film with 0.15% of MB–CaCO_3_ was slightly higher than the 0.1% of C–CaCO_3_ and lower than the control (Figure 5). Films filled with inorganic materials appeared to slow down the rate of film deterioration and biodegradation due to the strong interaction between filler and matrix, which is in agreement with [44] and [45]. When comparing the seaweed-based films with the conventional mulch film made of PLA, the conventional mulch film showed the lowest weight loss among all the films. This suggests that the rates of biodegradation for seaweed-based films are higher than the conventional PLA film. Although PLA is known to be biodegradable, the rate of biodegradation for mineralization to CO_2_ or CH_4_ can be lengthy, as microorganisms require adaption and induction of metabolic activity for the process of biodegradation [46]. Reference [47] reported that the biodegradability of PLA is usually hydrolyzed into low molecular weight oligomers before mineralization takes place. Many studies have been conducted to facilitate the attachment of microorganisms on the surface of the PLA film by incorporating additives or fillers or blending with lactic acid to accelerate the rate of biodegradation for PLA film [48,49].

**Figure 6:**
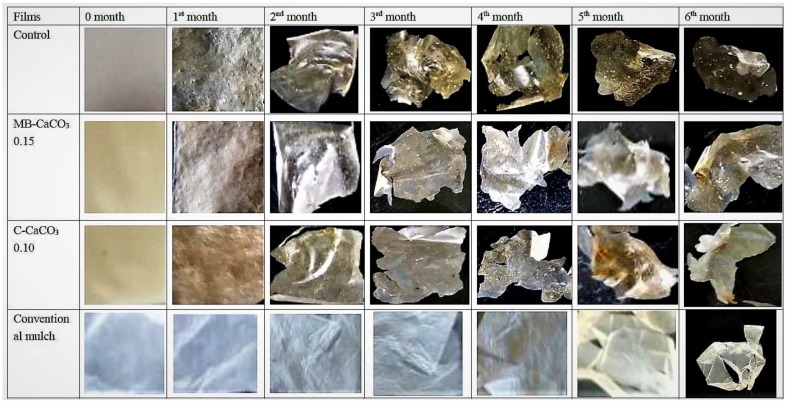
Changes in the appearance of films after 6 months of soil burial.

Besides weight loss in percentage, the changes of the films during soil burial can be distinguished by the changes in appearance shown in Figure 6. It can be observed that seaweed-based films began to change in color tone and surface structure as early as the first month. The changes were obvious from the second month onwards. Seaweed-based films started to shrink in size and cracked. From the third month up to the sixth month, seaweed-based films began to break down into smaller sizes, exhibiting irregular shapes. However, the conventional film made of PLA did not show any difference in size, surface structure, or color tone until the third month, where slight cracks can be observed. A drastic change of appearance can be noticed during the fifth and sixth months, where more cracks were observed. These observations were in agreement with Figure 5, where it was clearly shown that the degradation rate of fabricated seaweed-composite films was higher than conventional mulch film. 

## 4. Conclusions

This study produced seaweed-based composite films incorporated with C–CaCO_3_ and MB–CaCO_3_ and compared the physical, mechanical, structural, and biodegradability potential of seaweed-based films with the conventional biodegradable mulch film. The results indicated the incorporation of C–CaCO_3_ and MB–CaCO_3_ improved the properties of neat seaweed film. A composite film with C–CaCO_3_ exhibited superior mechanical properties and thermal stability, while composite films filled with MB–CaCO_3_ showed better physical properties. In general, seaweed-based films were comparable or even better than the conventional biodegradable mulch film in terms of mechanical properties, contact angles, biodegradability, and aesthetic value. The seaweed polymer film incorporated with MB-CaCO_3_ showed the highest contact angle of 100.94°, whereas conventional biodegradable mulch film showed a contact angle of 90.25°. FTIR studies revealed that there was a strong interaction between seaweed and CaCO_3_, which was further supported by the enhancement of the physical and mechanical properties of the film when CaCO_3_ was incorporated. The water vapour permeability of MB–CaCO_3_ based seaweed films was low (2.05 ± 1.06 g·m/m^2^·s·Pa) when compared to conventional mulch film (2.68 ± 0.35 g·m/m^2^·s·Pa), which makes the fabricated film as an excellent candidate for plasticulture application. It was found that the seaweed-based film incorporated with commercial CaCO_3_ exhibited a higher tensile strength, which was 84.92% higher than the conventional mulch film. SEM images of the fractured surfaces of the fabricated films revealed the strong interaction between seaweed and fillers. Moreover, water vapor permeability of the fabricated MB–CaCO_3_-based and C–CaCO_3_-based seaweed films decreased compared to control seaweed film and conventional mulch film, which was due to the induced hydrophobicity on the fabricated film during the incorporation of MB–CaCO_3_ and C–CaCO_3_ fillers. Overall, seaweed-based films showed remarkable results in physical, mechanical, thermal, barrier, and biodegradability properties compared to the conventional biodegradable films, indicating they can be a good candidate for plasticulture and agricultural application, serving a dual purpose as a plasticulture and fertilizer.

## Figures and Tables

**Table 1:** Physical and mechanical properties of seaweed-based films and conventional mulch film.

Samples	Thickness (µm)	WVP (×10^−10^ g·m/m^2^·s·Pa)	Tensile strength (N/mm^2^)	Young Modulus (MPa)	Elongation at break (%)
**Control (no filler)**	58.20 ± 25.03 ^b^	3.87 ± 0.05 ^c^	38.32 ± 2.99 ^b^	182.87 ± 35.88 ^a^	23.26 ± 2.77 ^b^
**MB–CaCO_3_** **0.15%**	60.00 ± 24.37 ^c^	2.05 ± 1.06 ^a^	58.50 ± 5.95 ^c^	286.79 ± 39.92 ^b^	29.1 ± 1.88 ^c^
**C–CaCO_3_** **0.10%**	61.20 ± 24.63 ^d^	2.45 ± 0.05 ^b^	69.31 ± 0.74 ^d^	351.89 ± 12.71 ^c^	25.22 ± 1.08 ^bc^
**Conventional mulch**	13.4 ± 1.14 ^a^	2.68 ± 0.35 ^c^	10.45 ± 1.49 ^a^	159.30 ± 17.86 ^a^	15.66 ± 3.72 ^a^

Values are represented as mean ± standard deviation. Means in the same rows followed by the same letters denote no significant difference (*p* < 0.05). Different letters (a, b, c, d), in the same column indicates significant differences (*p* < 0.05).

**Table 2:** Comparison of physical and mechanical properties of some seaweed-based films for packaging applications.

Types of Matrices	Fillers/Additives	Thickness (µm)	Water vapor permeability (WVP) (×10^−10^ g·m/m^2^·s·Pa)	Tensile Strength (N/mm^2^)	Young Modulus (MPa)	Elongation at Break (%)	References
Seaweed	Cellulosic pulp fiber	74–171	ND	45–81	ND	2.5–5.4	[13]
Seaweed	Microcrystalline Cellulose (MCC)	80–15	ND	20.21–29.76	ND	13.57–19.17	[33]
Seaweed/starch	none	58.7–145.1	1.73–4.27	41.37–65.73	1.64–6.43	6.17–18.4	[34]
Seaweed	Oil palm shell nanofiller	79.1–89.0	ND	31.4–44.8	2150–3000	2.08–3.30	[35]
Seaweed	Neem leaves	93–112	4.42–9.37	34.55–39.95	ND	17.64–20.73	[36]
Seaweed	MB–CaCO_3_ 0.15%	60.00 ± 24.37 ^c^	2.05 ± 1.06 ^a^	58.50 ± 5.95 ^c^	286.79 ± 39.92 ^b^	29.1 ± 1.88 ^c^	This work
Seaweed	C–CaCO_3_ 0.10%	61.20 ± 24.63 ^d^	2.45 ± 0.05 ^b^	69.31 ± 0.74 ^d^	351.89 ± 12.71 ^c^	25.22 ± 1.08 ^bc^	This work

ND = Note Determine. The values of current work are represented as mean ± standard deviation. Means in the same rows followed by the same letters denote no significant difference (*p* < 0.05). Different letters (a, b, c, d), in the same column indicates significant differences (*p* < 0.05).

**Table 3:** Color and opacity of seaweed-based films and conventional mulch film.

Types of Films	L*	a*	b*	C*	Opacity
Control	87.04 ± 0.04 ^a^	−0.18 ± 0.02 ^d^	17.51 ± 0.07 ^c^	17.51 ± 0.07 ^c^	15.31 ± 0.01 ^a^
MB–CaCO_3_	89.79 ± 0.08 ^c^	−0.63 ± 0.07 ^b^	14.29 ± 1.12 ^b^	14.3 ± 1.12 ^b^	16.21 ± 0.02 ^b^
C–CaCO_3_	88.34 ± 0.21 ^b^	−0.42 ± 0.05 ^c^	16.80 ± 0.39 ^c^	16.80 ± 0.39 ^c^	16.84 ± 0.13 ^b^
Conventional mulch	92.79 ± 0.10 ^d^	−0.82 ± 0.02 ^a^	5.48 ± 0.05 ^a^	5.54 ± 0.05 ^a^	6.34 ± 0.76 ^c^

(Different letters (a, b, c, d), in the same column indicates significant differences (*p* < 0.05).

**Table 4:** Thermal analysis of seaweed-based films and conventional mulch film.

Types of filler	*T*_on_ (°C)	*T*_max_ (°C)	Weight Loss due to Degradation (%)
Control	210	226	85
MB–CaCO_3_ 0.15%	226	249	80
C–CaCO_3_ 0.1%	236	257	83
Conventional mulch	260	352	95

**Table 5:** Functional groups, bonds, and vibrations of seaweed-based films and conventional mulch.

Wave Numbers (cm^−1^)			Functional Groups/Bonds/Vibrations
Types of Films	Before Burial	After Burial	
**Control**	3329	3325	O–H stretching vibration
	2924	2908	Stretching of aliphatic C–H
	1647	1647	Stretching vibration of C=O
	1219	1219	Sulphate ester group/ stretching vibration of S=O
	1034	1033	Stretching of C–O–C glycosidic bond
	922	922	3, 6-anhydro-d-galactose
	845	845	d-galactose-4-sulphate
**MB–CaCO_3_**	3325	3320	O–H stretching vibration
	1646	1616	Stretching vibration of C=O
	1219	1201	Sulphate ester group/ stretching vibration of S=O
	1034	1026	Stretching of C–O–C glycosidic bond
	922	902	3, 6-anhydro-d-galactose
	845	823	d-galactose-4-sulphate
**C–CaCO_3_**	3320	3302	O-H stretching vibration
	2889	2873	stretching of aliphatic C–H
	1643	1639	Stretching vibration of C=O
	1219	1253	Sulphate ester group/ stretching vibration of S=O
	1034	1014	Stretching of C–O–C glycosidic bond
	922	925	3, 6-anhydro-d-galactose
	845	848	d-galactose-4-sulphate
**Conventional mulch**	2959	2927	stretching of aliphatic C–H
	1712	1710	Stretching vibration of C=O
	1386	1386	CH deformation and asymmetric
	1361	1361	CH deformation and asymmetric
	1016	1012	C–O–C asymmetric
	699 to 729	679 to 723	Stretching vibration of C=O
